# A Spread Willingness Computing-Based Information Dissemination Model

**DOI:** 10.1155/2014/680421

**Published:** 2014-07-10

**Authors:** Haojing Huang, Zhiming Cui, Shukui Zhang

**Affiliations:** ^1^Institute of Intelligent Information Processing and Application, Soochow University, Suzhou 215006, China; ^2^Department of Computer Information Engineering, Guangdong Technical College of Water Resource and Electric Engineering, Guangzhou 510635, China; ^3^State Key Laboratory for Novel Software Technology, Nanjing University, Nanjing 210093, China

## Abstract

This paper constructs a kind of spread willingness computing based on information dissemination model for social network. The model takes into account the impact of node degree and dissemination mechanism, combined with the complex network theory and dynamics of infectious diseases, and further establishes the dynamical evolution equations. Equations characterize the evolutionary relationship between different types of nodes with time. The spread willingness computing contains three factors which have impact on user's spread behavior: strength of the relationship between the nodes, views identity, and frequency of contact. Simulation results show that different degrees of nodes show the same trend in the network, and even if the degree of node is very small, there is likelihood of a large area of information dissemination. The weaker the relationship between nodes, the higher probability of views selection and the higher the frequency of contact with information so that information spreads rapidly and leads to a wide range of dissemination. As the dissemination probability and immune probability change, the speed of information dissemination is also changing accordingly. The studies meet social networking features and can help to master the behavior of users and understand and analyze characteristics of information dissemination in social network.

## 1. Introduction

Currently, social networking platform based on personal relationships is increasingly welcomed by the majority of Internet users and businesses; the rapid development of foreign Twitter, Facebook, inland Microblog of Sina, Renren, Wechat of Tencent, and other social networking sites, showing the scale of the outbreak of the user growth, has a large number of users. It is reported that the number of users of Facebook has exceeded 750 million, and every day there are at least about 50% of users who log on Facebook. In March 2010, the network traffic of Facebook accounted for 7% of the network traffic of USA, and this ratio has exceeded the traffic of Google. CNNIC estimated that, at the end of 2009, the number of Internet users in China using dating sites and social networking sites has reached 124 million. Social network provides users with a platform to exchange information but also has some social features as follows: social network for the development of e-commerce has brought opportunities, government agencies can collect information for the formulation of a policy through social networks, and consumers can comment through social networks on a brand or products. With the development of Tablet PC, and smart phone applications, social networks based on portable devices became popular, such as Wechat of Tencent and Fetion, and its popularization and development were very fast.

Judging from the category of social networking, Facebook and Twitter represent two different kinds of social networks. Facebook and Wechat of Tencent are based on the strong relationship between friends to help maintain and improve friendship between friends; Twitter and Microblog of Sina are based on one-way and weak relationship, so that the network can help shape opinion leaders and news dissemination. Whatever the kinds of social network, every day it produces large amounts of user data and has an unprecedented scale, in the mass cluttered data, and contains a large number of valuable information. The large number of large-scale, emotional and groups of data generated on social networks, and continue to be spread, including text, voice, video and images, and so on. These data attracted experts and scholars of computer science, psychology, sociology, journalism, and other fields to study and explore, hoping to find more human unknown law by stronger social network data analysis and processing capacity.

The rapid development of social networks not only provides a new platform for the dissemination and sharing of information but also has become an important way to show users of self-worth, expressing interest demands and maintain interpersonal relationships. In social networks, everyone is an independent media. The information dissemination of social networks is different from traditional media which rely on content as form of the communication theme, and more dependent on the spreader's influence and social relations, through interest, relationship circles of friends or fans to spread information in a social network. This information will be seen by friends and fans, being shared and forwarded with a certain probability. The main reason for dissemination of information is that friends have common interests or good social relations and trust, which will achieve spontaneous diffusion and extension of information and continue to expand the impact of information. The study of evolution of social network, characteristics of user's behavior, information dissemination mechanisms, and potential dissemination law is having important theoretical significance and value of commercial applications.

## 2. Research on Dissemination Model of Social Network

Taking into account the large-scale and complex topologies and security issues of social networks, to directly research and analyze social networking platform are very difficult. Thus, the researchers tried to make the topology of the social network to be model abstraction according to key features and data of the real network to make topology model replace the social network to be researched by understanding the basic properties of topological model of social networks. Meanwhile, research on social network topology modeling in favor of deeply understanding of human's information exchange process.

Modeling is time-honored way to research network structure and behavior. Back in the 1960s, Paul Erdos and Alfred Renyi proposed the use of random graph theory to analyze the complexity of the network topology and the model is called “ER model.” In 1998, Watts and Strogatz presented “small world network” model, that is, WS model published in Nature; the main contribution of this model is presented in small-world networks between regular network and random network and can adjust by rewiring probability *p*, thereby allowing transformation of network structure between the rules and the random network; after Falatous presented degree distribution having the characteristics of the power-law distribution of the Internet, scale-free networks have become the main target being researched (Figure 2).

Information dissemination is also process of a kind of node link prediction. Paper [1] proposed that the greater similarity of two nodes led to a possibility of a link between two nodes. Liben-Nowell and Kleinberg proposed definition of similarity method based on network topology and these indicators were divided into two categories based on paths and based on nodes and analyzed a number of indicators for linked prediction results to the coauthor network in [2]. Clauset et al. considered that the connection can be seen as a reflection of the inherent hierarchy; they envisaged a maximum likelihood estimation algorithm to predict the link on the basis of paper [3].

In general, the traditional dissemination theory is often built based on linear thinking, the entire dissemination process is broken down into different parts rationally and accurately, and then abstract a simple dissemination mode. However, compared to the abstract pattern, actual dissemination process is much more complicated, and, disorderly, the dissemination results are not determined; spread behavior is unpredictable in social network. To understand the mode of dissemination and features of* SNS* better, this paper introduces the dynamics of infectious diseases and complex network theory.

### 2.1. Dynamics of Infectious Diseases

Epidemiological studies have a long history; infectious diseases dissemination model is an important method for theoretical studies on epidemic law of infectious disease. The classic models of infectious diseases dissemination models are* SIS* and* SIR* models. In* SIS* model, individuals within populations are only two typical states: *S* (susceptible) and *I* (infected). In contact with an infected individual, there will be a certain probability of healthy individual change into infected individual or infected individual will recover to health. The so-called* SIR* model refers to each individual as only one of three states: *S* (susceptible status), *I* (infection status), and *R* (removed status). In contact with an infected individual, there will be a certain dissemination probability of a healthy individual change into infected individual, with a certain probability of infected individual change to removed status. After that, on the basis of* SIS* model and* SIR* model, scholars have proposed more realistic disease dissemination model.

### 2.2. Complex Networks

Complex network is a method and angle to study complexity of the system and is the basic framework of a complex system. Xuesen Qian made a more stringent definition for the complex network: a self-organizing, self-similar attractor, small world, some or all nature of scale-free network is called a complex network. Social networking is based on the theory of six degrees of separation established, small-world and scale-free nature, which are described from different angles complex networks has a distinct characteristic, so the networks that individual nodes formed can be seen as a kind of complex network.

With the rapid development of complex network theory, the scientists found that in real life many networks have a small world, scale-free characteristics, and network topology structure plays an important role in the dissemination. Unfortunately, in the last study, the scientists put all the focus on the discussion of the rule of spread but did not give due attention to the network topology. Thus, scholars began to study diseases, computer viruses, and rumors spread in the small world and scale-free networks formed on the dissemination dynamics of complex networks. DJ Watts and SH Strogatz simulate the spread of disease in the simple world network; the study found that disease spread in the small-world network faster and easier than in the rule network. R. Pastor-Satorras and A. Vespignani studied the* SIS* model of the disease spread on scale-free networks and found that dissemination threshold does not exist, which means that even a very small intensity of dissemination of disease is also enough to make disease spread in network. This conclusion has changed many traditional theories of diseases spread that only when the dissemination rate exceeds a threshold value, large-scale dissemination is possible.

Weng et al. found that follower and friends of Twitter users obeyed a power law distribution in [4]. Cha et al., who have found the opposite conclusion, believed in the existing weak correlation between Twitter user's friends and fans in [5]. Grabowski found user nodes of which small degree obeys a power law distribution and user nodes of which large degree obeys exponential distribution, and the number of community in network obeys a power law distribution in [6]. Ding et al. in [7] found the following conclusions: involvement characteristic of user and length of replies on the posts were subject to a power-law distribution, but the number of read posts did not obey a power law distribution.

Scholars made various studies on the dissemination phenomena in complex networks, such as the rumors dissemination in [8], the new products dissemination in [9], and the spread of disasters. DH Zanette simplified the complex mechanisms of the spread of rumor and used* SIR* model to study the rumors spread of small-world network. Moreno et al. pointed out that, in the dissemination of rumors in the nonuniform network, the final number of people who heard rumors but do not spread rumors was closely related to the probability of infection, regardless of the dissemination source. Candia et al. studied the effects of the mass media and noise on cultural dissemination, pointing out that the social impact is an important factor in designing a successful advertising campaign. PS Dodds et al. proposed a model to promote the phenomenon has spread, will be exposed as a historical memory is introduced into the model to study the effects of infection. In addition, in the field of the marketing and promotion of new products, most studies suggested that a small number of influential individuals play important role in the dissemination of information and opinion formation process. But Watts and Dodds put forward a different point of view; they think of large-scale spread not by pushing influential individuals but by a large number of groups easy to be affected around influential individuals in [10].

The dissemination dynamics of complex network set some rules for the system to allow system evolution spontaneously under certain circumstances, and then observe the evolution of certain properties of the system. Therefore, the spread model reflects the thinking of complex network modeling. The research on the dissemination law of all kinds of information (such as infectious diseases, computer viruses, information, etc.), grasp of the effective methods to control its spread in complex networks, it is a difficult and meaningful work.

## 3. Information Dissemination Model

### 3.1. Dissemination Mechanism and Process

In social networks, at some point, user *A* finds update dynamic information of their friends user *B* and then user *A* may share or forward. This behavior will appear in the dynamics of friends of user *A*, so they can see these messages. Next, a friend of user *A* and a friend of user *A*'s friend may have a similar operation with user *A*, which constitutes the information dissemination process. If user *A* is not interested in the message user *B* sent so did not make any response, then user *A*'s friends did not see this message, and the information dissemination process may be interrupted. In addition, user *B* generally has a number of friends, and other friends also have similar behavior as *A*'s. And so on, the information spread in the form of a mesh-like around *A*. When a user sends a message, all his friends are likely to share and forward the message or browse message without operation in a certain probability.

Based on the above analysis of the dissemination mechanism of social network information dissemination processes, the following can be summarized.

1. User *v* sends news.
2. *v*'s any friends, *u*, can alert a new message through the system or visit *u*'s space and other ways to learn the message (the model does not consider the specific behavior of the user, assuming that all of *b*'s friends will be informed with the message in the probability *p*).
3. If *u* is interested in the message of the friends, the message will be forwarded and form secondary dissemination of messages, but in forwarding behavior usually only once. Otherwise, the user will become immune to those information that will not be disseminated.
4. User *u* is changed into the role of the main object spread information and repeats processes (2) and (3) until no user forwards the message.

### 3.2. Dissemination Rules

In summary, in the* SNS* network, the user releases news which will be seen by his friends and shared and spread in a certain probability. Also, if his friends do not agree with its content or are not interested in it, they will not spread. In this paper, a user is defined as a node in* SNS* network, a friend's relationships between individuals can be used abstractly to represent the edges between nodes, only the information along the edges.

According to information dissemination rules in* SNS* network, nodes are divided into three categories: *S* (susceptible node), *I* (infected node), and the *R* (removed node). *I* (infected node) represents the node that has accepted information from its neighbor nodes and has ability to disseminate the information. *S* (susceptible node) indicates that the node did not accept the information from its neighboring node but has chance to spread information; the probability of infection exists. *R* (removed node) indicates the node which has contacted with the neighbor node's information but does not have the ability to disseminate. Dissemination of information is human's an initiative act of spreading information not a passive process that the person is infected by virus. This process is impacted not only by subjective factors but also by objective environment. Thus, the status of nodes changes between *S* (susceptible node), *I* (infected node), and the *R* (removed node) which depends not only on the status of itself but also the status of its associated neighbor nodes; the following dissemination rules can be defined.

1. In the model, in each initial process of information dissemination, there is only one source node spreading information, and the link degree is *w*.
2. If a susceptible node is in contact with an infected node in a certain time step *T*, the susceptible node will become infected node with a probability dissemination *θw*.
3. If an infected node is in contact with a removed node, the infected node will become removed node in probability *ωw*, and the dissemination of information stops in this time.
4. The dissemination of information does not last endlessly down and will stop in certain velocity *v*, and without contact with other nodes.

### 3.3. Information Dissemination Model

The information dissemination model consists of infected nodes and information dissemination model circle, designing the mean field equations based on infectious disease dynamics and complex network theory. The information would be shared or forwarded by nodes at different speeds. The information is generally forwarded when node is contact with the information the first time, based on when nodes contact with information. In the information dissemination process, the spread wishes of the nodes will affect the evolution of the entire dissemination, which consists of the strength of the relationship, the degree of node identity, and the contact point of the frequency components.

#### 3.3.1. Definition

Supposing *S*, *I*, *R* is the status of susceptible node, infected node, and removed node, respectively, *k* nodes in the network which the degree is *k* at time *t*. For easy description of the model, introduce the set *P*_*t*_(*v*) and collection of *N*_*t*_(*v*), *P*_*t*_(*v*) represents set in the status of the node *I* in the time step *t*:

*P*_*t*_(*v*) = {*v*∣*v* ∈ *V*, the  status  of  *v*  is  *I*}.

*N*_*t*_(*v*) represents the set of the adjacent nodes of which status of *v* is *S* in the time step *t*:

*N*_*t*_(*v*) = {*u*∣(*v*, *u*) ∈ *E*, the  status  of  *u*  is  *S*}.

This process is gradually advancing forward based on the time step *t* (time-step) as a unit, and the information dissemination process is as follows.

1. At the time of step *t* = 0, randomly select a node *v*_0_ as an initial node spread message, that is, *P*_*t*_(*v*) = {*v*_0_}, and the degree of the node *v*_0_ is denoted by *k*_*v*_0__.
2. When friends of *v*_0_ node contact with information, *n* nodes become the dissemination status, sort in accordance with the time when message may be forwarded or shared, and put them in the dissemination queue *Lv*_01_{*v*_01_, *v*_02_, *v*_03_, *v*_04_, *v*_05_, *v*_06_,…, *v*_0*n*_} of *v*_0_. Similarly, it will produce friends dissemination queue *Lv*_02_ … *Lv*_0*n*_ of *v*_01_, *v*_01_,…, *v*_0*n*_.
3. When dissemination be to *t* = *n*  (*n* = 1,2, 3…), each node *v* belonging to the set *P*_*t*_(*v*), put nodes into the collection *P*_*t*_(*v*) from the collection *N*_*t*_(*v*), and then reset the status of *v* to *R*, and *v* is removed from the set *P*_*t*_(*v*).
4. When the set *P*_*t*_(*v*) is empty, go to the next time step *t* + Δ*t* and repeat the previous steps for the collection *P*_*t*_(*v*) until no new node is activated; the dissemination process is finished.

#### 3.3.2. Dissemination Circle

The dissemination range which let infected nodes be the center is called a dissemination circle. The sideline between the infected nodes is called dissemination path in dissemination circle. The rapid information dissemination will connect with many dissemination circles in the network. Dissemination circle is method to build the information transmission model; it is mainly used for building and explaining the process of information transmission.

Due to the fact that the information dissemination is not necessarily unique, that is, in a social network infected nodes may appear and almost simultaneously began to spread information, taking into account that the nodes will continue to increase or demise, according to multiple LAN world model MLW, suppose there is *n* independent dissemination circles in a social network, and at least one infected node in each circle inside, *n*′ nodes and *s*′ edges, do the following.

(1) In order to define the nodes and edges increased in social network, increase a dissemination circle which consists of *n*′ nodes and *s*′ edges in probability *p*.
(2) With probability *q* to add a new node to the dissemination circle that already exists, create S1 edges with nodes of the same dissemination circle. Randomly select a dissemination circle *Q* in network and the probability of a new node to connect the nodes as below, and repeat this process S1 times. With probability *r* to increase S2 edges to a selected circle, select one of the two endpoints of the edge of a circle which is randomly selected as infected node, and the probability of another is calculated for S2 times repeatedly according to the following formula:
  (1) ∂(ki)=ki+a∑j∈Q(kj+a).
(3) In order to define the demise of nodes and edges in social circles, with probability *s*, reduce m1 edges inside the circle, one of the two endpoints of the edge of a circle is randomly selected as infected node, and selection method of another node is *N*(*t*) is the number of nodes within a circle; calculate repeatedly for m1 times:
  (2) $$\begin{array}{c} \partial^{\prime }\left(k_{i}\right)=\frac{\left(1-\partial \left(k_{i}\right)\right)}{N_{Q}-1}. \end{array}$$
(4) With the probability *I* in a selected dissemination circle and other dissemination circles with m2 edges, a circle is selected randomly, and select a node as an endpoint of edge based on the probability formula; the other endpoint inside another circle is randomly selected and also selected according to the probability formula; calculate repeatedly for m2 times.

The parameter satisfies 0 < *q* < 1, 0 ≤ *p*, *r*, *s*, *u* < 1, *p* + *q* + *r* + *s* + *i* = 1.

#### 3.3.3. Evolution

When a node who becomes the first infected node release information. The nodes in friends circle of the first infected node are in contact with information; part of them become infected nodes, and the rest become removed nodes. Because the removed nodes will make information dissemination stop, it is assumed that a node *u* at time *t* is not infected status, *p*_*s*_ is probability that node within period [*t*, *t* + Δ*t*] is in uninfected status, *p*_*si*_ is probability that susceptible node becomes infected node and *p*_*si*_ = 1 − *p*_*s*_, and

(3) $$\begin{array}{c} p_{s}={\left(1-\Delta t\theta w\right)}^{lv}. \end{array}$$

*lv* = *lv*(*t*) represents the dissemination wishes value of the node *u* at time *t*, which will be discussed in the next section. *p*_*ss*_ is probability that the node becomes infected node within time [*t*, *t* + Δ*t*], the probability of the node becomes removed node *p*_*sr*_ = 1 − *p*_*ss*_, and

(4) $$\begin{array}{c} p_{ss}={\left(1-\Delta tw\right)}^{1-lv}. \end{array}$$

Assuming *N*(*w*, *t*) as the total number of nodes whose degree is *w* at time *t*, *I*(*w*, *t*), *S*(*w*, *t*), *R*(*w*, *t*) at time *t*, respectively, and as the total number of infected node, susceptible node, and removed node whose degree is *w*, then

(5) $$\begin{array}{c} I\left(w,t\right)+S\left(w,t\right)+R\left(w,t\right)=N\left(w,t\right). \end{array}$$

Since the susceptible nodes in the network will become infected nodes with a certain probability, then the number of infected nodes will continue reducing; therefore, the changes of the number of susceptible nodes whose degree is *w* in the time *w*  [*t*, *t* + Δ*t*] are as follows:

(6) $$\begin{array}{c} S\left(w,t+\Delta t\right)=S\left(w,t\right)-S\left(w,t\right)\left(1-p_{ii}\left(w,t\right)\right). \end{array}$$

A part of the susceptible nodes will become infected nodes, so the number of infected nodes will increase but some become will removed nodes, thus reducing the number of infected nodes. The changes of the number of infected nodes whose degree is *w* in the time *w*  [*t*, *t* + Δ*t*] are as follows:

(7) I(w,t+Δt)=I(w,t)+S(w,t)(1−pii(w,t)) −I(w,t)(1−pss(w,t)).

The number of removed nodes will increase because infected nodes will become removed nodes. The changes of the number of removed nodes whose degree is *w* in the time *w*  [*t*, *t* + Δ*t*] are as follows:

(8) $$\begin{array}{c} R\left(w,t+\Delta t\right)=R\left(w,t\right)+I\left(w,t\right)\left(1-p_{ss}\left(w,t\right)\right). \end{array}$$

Let *i*_*w*_(*t*), *s*_*w*_(*t*), *r*_*w*_(*t*), respectively, be the ratio of infected nodes, susceptible nodes, and removed nodes of a social networking whose degree is *w*. *θw* is the probability of susceptible nodes which are infected when contacting with infected nodes, *ωw* is the probability of infected nodes which recover when connecting with removed nodes.

In the nonuniform power-law distribution network, design the mean-field equations to describe the changes in three types of nodes as follows:

(9) dsw(t)dt=[(1−Δtθw)lv−1]sw(t)∑w′w′P(w′)iw′(t)〈w〉.diw(t)dt=[1−(1−Δtθw)lv]sw(t)∑w′w′P(w′)iw′(t)〈w〉 −[1−(1−Δtωw)1−lv]iw(t) ×∑w′w′P(w′)[iw′(t)+rw′(t)]〈w〉,drw(t)dt=[1−(1−Δtωw)1−lv]iw(t) ×∑w′w′P(w′)[iw′(t)+rw′(t)]〈w〉.

**Figure 1:**
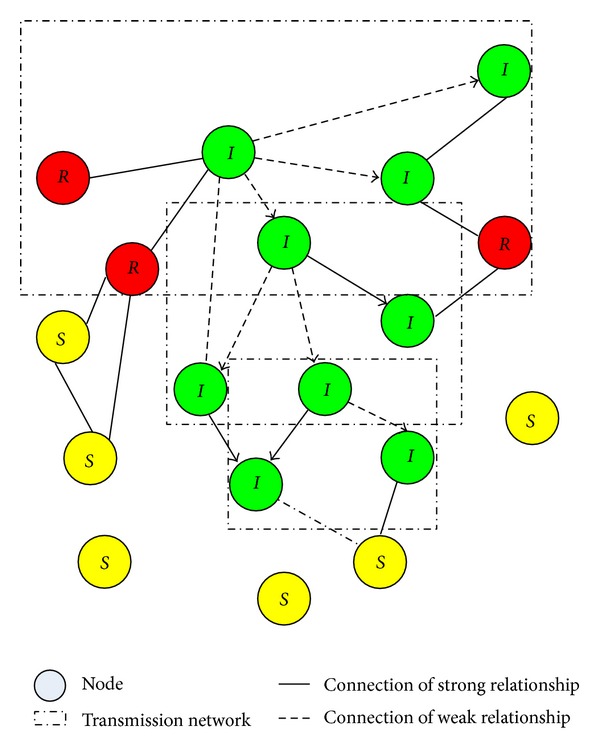
Evolution of nodes in process of information dissemination.

**Figure 2:**
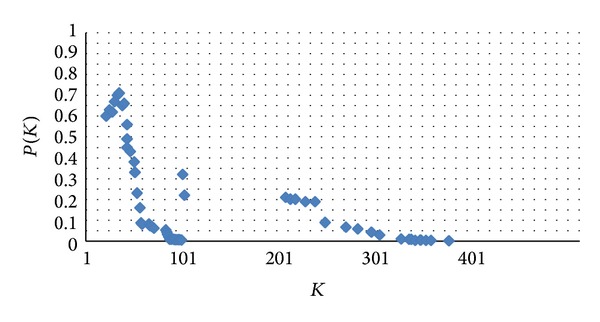
Initial distribution of degree.

Evolution of nodes in information dissemination model as shown in Figure 1.

## 4. Will of Dissemination Computing

Because the main cause of forwarding information is user's wishes. Will of dissemination is attributed consists of three factors: the strength of relationship between nodes, the identity views of nodes to information (interest similarity), the frequency of susceptible node is contact with infected node.

The strength of the relationship is often understood as the basis for the information dissemination, whether the stronger relationship will be a higher probability of information dissemination. According to the study, a strong relationship between the nodes produces a closed view space, leading to dissemination probability which is lower than weak ties nodes. Zhao et al. pointed out that the weak links in the social networks have subtle effects. On the one hand, the weak connection is a bridge connection between isolated communities; when gradually removing weak connection, information transmission coverage will decrease sharply. On the other hand, weak connection cannot speed up the information transmission, instead of randomly selected connection which can achieve this purpose in [11].

Some scholars focus on exploring the topic of interest degree of [12], the number of users' comments of [13], and the number of discuss a topic impact on the topic spread in [14–16]. Martins et al. in [17] also found in the case of the introduction of the third kind of neutral point of view, the individuals hold a neutral point of view which would be in the middle between two opposing views; when most of individuals without comment at first, the individuals express views were not be advised to strengthen at most of the time, the degree of extremism happen could be weakened. The identity views to information between friends come from the similarity social roles; social roles overlap as high as the probability of identity views to information.

In the social network, the source of the information is not necessarily the only one node; for example, while a number of friends forwarded the same news “serious violent incidents occurred in Kunming” in circle of friends, the frequency of contact with information can also be a factor affecting the information dissemination. Furukawa et al. studied blog users' reading behavior, including references, links, and comments, and found that 50% of the posts had been repeatedly read, and the existing 20% chance is read by the new login users in [18]. Therefore, calculate the three factors to get consolidated determination of information dissemination will of nodes:

(10) $$\begin{array}{c} lv\left(t\right)=\overset{Q}{\underset{i}{\sum }}\frac{P\left(X\;|\;s_{j}=+1\right)P_{\text{contact}}\left(K\right)}{s_{ij}}. \end{array}$$

“*i*”, “*j*” are both susceptible nodes. In formula (10), “*Q*” means that the maximum number of susceptible nodes has contact information, “*i*” means that the minimum number of susceptible nodes has contact information at time *t*, *i* < *j* < *Q*. *s*_*ij*_ shows a strength of relationship between the nodes *i* and *j*, and *P*_contact_(*K*)*t* represents the probability of susceptible node *I* which was infected when was in contact with *K* infected nodes in time *t*. *P*(*X*∣*s*_*j*_ = +1) is the probability of the node *i* which thinks *X* is the best ideas when the node *i* found that the view of node *J* is *X*.

### 4.1. The Strength of Relationship between the Nodes

The strength of relationship between the nodes will affect information dissemination, and the strength of relationship between the *s*_*ij*_, *r*_*ij*_ shows the same number of nodes in adjacent node of *i* and *j* node, which means the number of mutual friends. *q*_*i*_ and *q*_*j*_ indicate that the degree *s*_*ij*_ of node *i* and node *j*, which is greater, the link between two nodes is stronger, whereas weaker:

(11) $$\begin{array}{c} s_{ij}=\frac{r_{ij}}{q_{i}-1+q_{yj}-1-r_{ij}}. \end{array}$$

### 4.2. The Identity Views of Nodes to Information

In social networks, the identity views or similarity of interest of nodes, basically, are directly proportional to similarity of roles. The more similar the role of two nodes is, the higher the probability of recognition of certain topics is. For example, node *A* and node *B* are friends; both in the role of social networks have a common father, educators, automobile owners, and football fans, so when the node *B* shares news about the 2014 soccer World Cup and a message about the stock market crash, the probability of the former will be forwarded by node *A* is larger.

Martins used Bayes' theorem to view individual's decision-making process modeling and proposed CODA (continuous opinions and discrete actions) model in [19]. The results of [20] showed that CODA model can restore the normal evolution of continuous or discrete viewpoint and be able to explain the mechanism of extremism. Martins et al. studied the CODA model emerged in the case of different topologies and found that strengthening the interaction between individuals can weaken the extremist trend in [21]. Martins et al. also studied the diffusion of new products in [22].

Modeling of view selection according to CODA model, supposing there are two competing viewpoints *A* and *B*, the view *S*_*i*_ of individual *i* is defined as a two-dimensional discrete variable, and *S*_*i*_ = +1 means that *i* chooses view *A*, showing that identity views or similar interest are high; *S*_*i*_ = −1 means that *i* chooses view *B*, showing that the low point of identity views or interest are not similar. Defined a priori probability *P*(*A*) is tendency of the individual i for view *A*, is the probability of individual *i* think “*A* is the best point of view”; probability *P*(*B*) is tendency of the individual *i* for view *B*, showing that individual *i* supports view *B*. Assuming *P*(*A*) = *p*_*i*_, then *P*(*B*) = 1 − *p*_*i*_ and *pi* ∈ [0, 1].

Meanwhile, the probabilities in the model are defined as follows: if *A* is similar interest or identity view, the interactive each other (refer to *i* and neighbor *j* of *i*) select *A*'s probability *α* = *P*(*s*_*j*_ = +1∣*A*). If possibility *B* is similar interest or identity view, the possibility that interactive each other select view *B* is *β* = *P*(*s*_*j*_ = −1∣*B*). Accordingly, −*α* = *P*(*s*_*j*_ = −1∣*A*), −*β* = *P*(*s*_*j*_ = +1∣*B*). Under normal circumstances, people usually choose the view they agree with, therefore setting the ranges of *α*, *β* as real numbers (0.5, 1]. In accordance with Bayes' theorem, when individual *i* seeing *j* held view *A*, the probability that *i* considers *A* is the best choice as follows:

(12) P(A ∣ sj=+1) =P(A)P(sj=+1 ∣ A)P(sj=+1) =P(A)P(sj=+1 ∣ A)P(A)P(sj=+1 ∣ A)+P(B)P(sj=+1 ∣ B).

Similarly, when individual *i* seeing *j* held view *B*, the probability that *i* considers *B* is the best choice as follows:

(13) P(B ∣ sj=+1) =P(A)P(sj=+1 ∣ B)P(sj=+1) =P(B)P(sj=+1 ∣ B)P(A)P(sj=+1 ∣ A)+P(B)P(sj=+1 ∣ B).

Define a priori ratio 0(*A*) as the ratio of the individual *i* supporting *A* and *B*; that is,

(14) $$\begin{array}{c} O\left(A\right)=\frac{p\left(A\right)+1}{p\left(B\right)-1}=\frac{P_{I}+1}{-P_{I}}. \end{array}$$

When seeing *J* held view *A*, the posterior ratio *O*(*A*∣ = *σ*_*j*_ + 1) that the individual *i* think *A* is the best view as flowing:

(15) O(A ∣ =σj+1)=P(A ∣ sj=+1)P(B ∣ sj=+1)=P(A)P(sj=+1 ∣ A)P(B)P(sj=+1 ∣ B)=PI+1−PI=α1−β.

### 4.3. The Frequency of Susceptible Node Contacting with Infected Node

When the number of infected nodes forwarding the same information increases surrounding nodes, it will lead to frequency of contact with the information increased, and the will to forward the information will be impacted. According to definition of promotion model, at each time *t*, node *i* is connected to the node *j* randomly. If the node *i* is susceptible node, *j* is the infected node, and the node *i* obtains a positive dose *d*_*i*_(*t*) with probability *p*, where each of *d*_*i*_(*t*) is subject to the distribution function *f*(*d*). Each individual retains total dose accepted during the last *T*:

(16) $$\begin{array}{c} D_{i}\left(t\right)=\overset{t}{\underset{t^{\prime }=t-T+1}{\sum }}d_{i}\left(t^{\prime }\right). \end{array}$$

When *D*_*i*_(*t*) > *d*_*i*_^∧^, susceptible node *i* becomes infected node. In the *T* period, probability that susceptible node is infected after which contact with *K* infected nodes as a result:

(17) $$\begin{array}{c} P_{\text{contact}}\left(K\right)=\overset{K}{\underset{k=1}{\sum }}\left(\begin{array}{c} K\\ k \end{array}\right)p^{k}P_{k}{\left(1-p\right)}^{K-k}. \end{array}$$

## 5. Simulation and Analysis

Unlike random scale-free networks that all nodes in the network have been identified, because of its status of dynamic growth, new nodes continue to be added, while these new nodes are not as random network and have the same probability of connection with other nodes. It has a greater probability of establishing a connection with those who have a lot of links to nodes, consistent with the characteristics of social networks, so the scale-free network model is suitable to do simulation and analysis. Classic *BA* scale-free network structure is from a network with *m*′ nodes, every time steps to add 80 new nodes, and they are connected with *m*  (*m* < *m*′) preexisting nodes, the size of the generated network is *N* = *t* + *m*′ after *t* step, and the total number of edges is *m*_*t*_.

### 5.1. Density of Different Nodes Over Time

Firstly, test the evolution of density of different nodes over time, the basic data of the network in Table 1.

**Figure 3:**
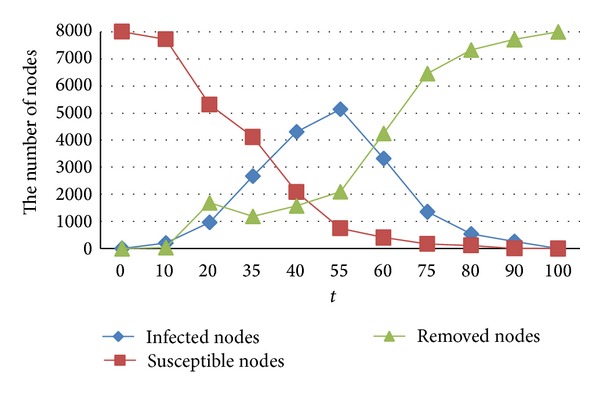
The change of quantity of nodes in the process of information dissemination.

Supposing there is only one infected node in the information dissemination at the beginning, the remaining nodes are all susceptible nodes. Secondly, the model parameters set is as follows: *θw* = 0.46, *ωw* = 0.23, *lv*(*t*) = 0.062, and the iterations are 500. Analysis of density evolution of infected nodes, susceptible nodes, and removed nodes is as shown in Figure 3.

Experimental analysis shows that the number of infected nodes in the initial stages of dissemination reached the maximum then decreased rapidly until tending to zero. The number of removed nodes showing an upward trend in the twists and turns, after a certain stage, tends to rise steadily until reaching the total number. Susceptible nodes continued to decay until the number of it goes to zero.

The following begins to study analysis of will of dissemination computing and the impact of each factor to the behavior of information dissemination.

### 5.2. Strength of the Relationship Impact on Information Dissemination

**Figure 4:**
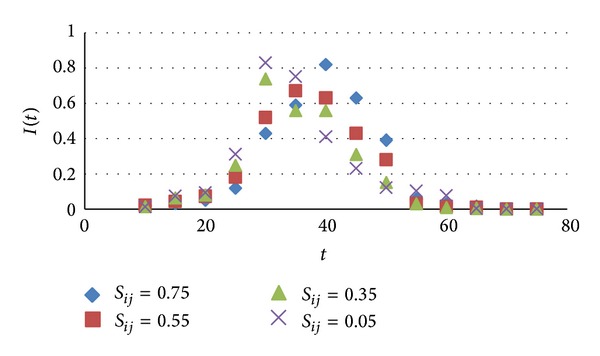
Relationship strength between nodes impact on information dissemination.

Studying the strength of the relationship between initial infected nodes and others infected nodes as *S*_*ij*_ impact on their spread behavior in this section. When under these four cases: *S*_*ij*_ = 0.75, *S*_*ij*_ = 0.55, *S*_*ij*_ = 0.35, *S*_*ij*_ = 0.05, the changes of density of infected nodes over time as shown in Figure 4.

As can be seen from Figure 4, when the relationship was weak between the nodes, in the evolution at the same time, density of infected nodes reaches a maximum within a short time; the rate of change was fast. When the relationship was strong, density of infected nodes also can reach high densities, the rate of change was slow, and then, which decay fast, the speed of decay was faster than weak ties nodes. But when the time tends to infinity, regardless of the relationship between the nodes, eventually density of infected nodes tends to 0 in network, as the longer, as the smaller the density changes caused by the strength of the relationship. This was due to social network having a high connectivity, longer time of dissemination, and greater range time of dissemination. Thus, a weak relationship between nodes in a short time with greater information dissemination capabilities and strong ties nodes can also cause a wide range of information dissemination when dissemination time was longer in the case, but its density of infected nodes decays faster than density of weak relations nodes. This test is in line with the situation of dissemination information in social network and also is an important feature of social networks. In real life, often our acquaintance published message, which was not quick to be shared or forwarded in a short period in the network and easy to form a closed space hinder information dissemination. But with the passage of time, this information can be gradually spread, but its dissemination cycle was very short. The information from some of the stars, the public or service account, can quickly be spread in network, and dissemination cycle was long, resulting in a greater social impact. This is consistent with the simulation in paper.

### 5.3. Identity Views Impact on Information Dissemination

**Figure 5:**
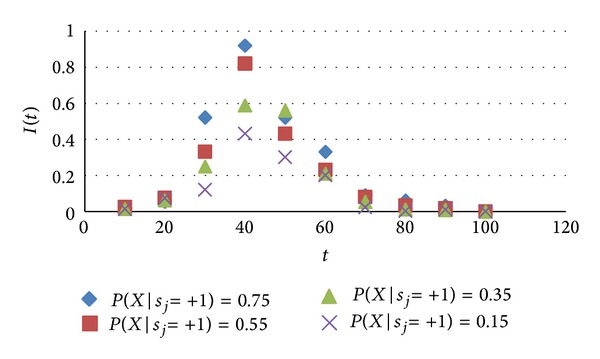
Identity views impact on information dissemination.

When susceptible node exposed to a certain point of view held by the infected node, analyzing situations of choosing to share or forward information. Supposing probability of *X* is considered the best view after node *i* saw the infected node *J* held view *X* is: *P*(*X*∣*s*_*j*_ = +1), which values were 0.15, 0.35, 0.55, 0.75. In social networks, the change of infected nodes with time is as shown in Figure 5.

As can be seen from Figure 5, the large probability of the best identity views, the mass density of nodes, and the fast increasing speed, when the density of infected nodes peaked, gradually tends to zero. But when the probability of the best identity views was low, density of infected nodes was low, the speed of it becoming removed node was faster than the speed of large probability. That is, when node *i* was exposed to node *j* which held view dissemination *X*, if the probability that X was considered the best view by *i* was large, the density of infected nodes increased significantly in a short period of time and slowly declines after reaching a maximum until the entire nodes became removed status in dissemination process.

### 5.4. Frequency of Contact Impact on Information Dissemination

**Figure 6:**
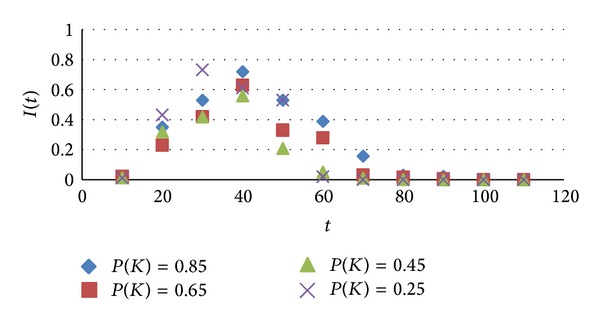
Frequency of contact impact on information dissemination.

Studying in the period *T*, susceptible nodes *I* were in contact with the *K* infected nodes, and thus the probability of being infected *P*(*K*), which's value are set: 0.25, 0.45, 0.65, 0.85. In social networks, the change of infected nodes with time is as shown in Figure 6.

As seen from Figure 6, before the density of infected nodes reached its maximum, the smaller *P*(*K*), so that the mass density of nodes rose faster, when the dissemination process is up to a certain time, density decreased rapidly until it tends to 0, dissemination is completed within a short period of time. After the density reached a maximum, when the probability *P*(*K*) was large, the times of density rise and fall were slower, and the dissemination time was longer. But different probability values impact on the maximum density difference was not obvious.

### 5.5. Infection Probability Impact on Density of Infected Nodes

**Figure 7:**
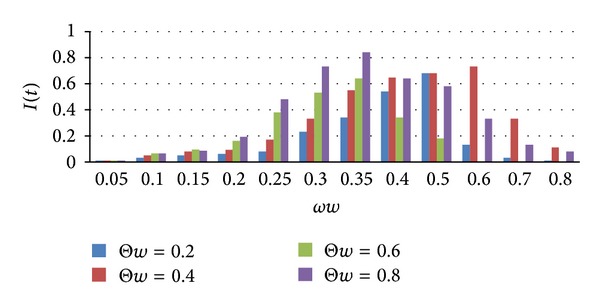
Dissemination probability impact on density of infected nodes.

Study impact on dissemination behavior in social networking with the variation of *ωw* when the probability of dissemination Θ*w* is different. Θ*w* is the probability of the susceptible nodes which is infected when encountered with infected nodes; set its values Θ*w* = 0.2, Θ*w* = 0.4, Θ*w* = 0.6, Θ*w* = 0.8. The change of density of infected nodes of these four cases over time is as shown in Figure 7.

As seen from Figure 7, the probability of infection was large, infected nodes increased fast, density was high, and its dissemination speed was much higher than the low probability. But Θ*w* = 0.8, and the speed of density of infected nodes decreased significantly which was slower than other forms of dissemination probabilities. This indicates that in social network, when the number of susceptible nodes reached maximum, the speed of which became removed nodes was slower, described the information in a period of time will remain strong dissemination state, and the dissemination range was greater. When Θ*w* = 0.2, the maximum value of the infected nodes was low, a longer time was required for the change, but the speed of density of infected nodes decreased faster, indicating that a low probability of infection will lead to a shorter duration of the dissemination status, and the probability and ranges of information dissemination were relatively small.

### 5.6. Immune Probability Influence on the Density of Removed Nodes

**Figure 8:**
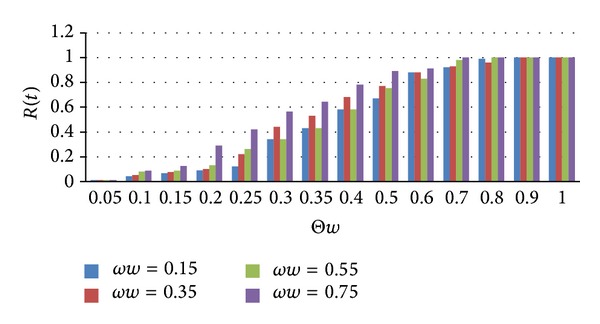
Immune probability influenced on the density of removed nodes.

Studies when the immune probability *ωw* is different values, the changes in the removed nodes and relationships Θ*w* impact on the spread of social networking behavior. *ωw* after immunization nodes spread across nodes become immune status of probability, and its value are *ωw* = 0.15, *ωw* = 0.35, *ωw* = 0.55, *ωw* = 0.75, which changes as shown in Figure 8.

As can be seen from Figure 8, the greater the immune probability, the faster density of removed nodes *r*(*t*) which increased, and longer times the status of information dissemination reach steady to be. Even when immune probability was great, information can still be spread in the network. However, information dissemination will be achieved and stabilized in a short time, i.e., in the dissemination of information existing a certain time delay. When immune probability increased, *r*(*t*) gradually increased. When immune probability was low, in early times of information dissemination, the proportion of the immune status increased more slowly and will increase rapidly after a certain stage until being steady.

## 6. Conclusion

By analyzing the form and the characteristics of mode of information dissemination in social networks, combined with dynamics of infectious disease and complex network theory, based on the improvement of the* SIR* model, a social network model of information dissemination was proposed, and differential evolution equations was constructed. The model defines the dissemination rules and procedures, as well as the will of the three factors which affect the dissemination behavior and calculation method. By constructing* BA* scale-free network, finished a social network simulation analysis of information dissemination process, and three different types of user's behavior and rule in the information dissemination. The experimental results showed that different degrees of nodes show the same change trend in the network, information dissemination threshold is almost zero, and a node of which the degree was small may lead to a large release of information dissemination even. Weak relationship between nodes, the higher the probability of view selection and higher frequency of contact with the information, lead to quickly and large-scale dissemination. However, the information dissemination could not be unlimited; when *t* tended to infinity, the information dissemination process would reach steady state, and the number of various types of nodes would not change; when dissemination probability and immune probability changed corresponding to change of the rate of information dissemination will occur. This paper helps to gain a deeper understanding of evolution of the social network and behavior of information dissemination. The next step of the research would be influenced of the nodes impact of information dissemination and evaluation methods on value of information.

## Figures and Tables

**Table 1:** Parameters of *BA* network.

The total number of nodes	5000
Average degree	11.28
Maximum degree	368
Minimum degree	0
Clustering coefficient	0.0688293
With the same coefficient	0.0053218
Time step *t*	100
Power-law exponent	1.5
